# Molecular Basis of Triple Negative Breast Cancer and Implications for Therapy

**DOI:** 10.1155/2012/217185

**Published:** 2011-11-02

**Authors:** Parvin F. Peddi, Matthew J. Ellis, Cynthia Ma

**Affiliations:** Division of Oncology, Department of Medicine, Siteman Cancer Center, Washington University School of Medicine, St. Louis, MO 63110, USA

## Abstract

Triple negative breast cancer is an aggressive form of breast cancer with limited treatment options and is without proven targeted therapy. Understanding the molecular basis of triple negative breast cancer is crucial for effective new drug development. Recent genomewide gene expression and DNA sequencing studies indicate that this cancer type is composed of a molecularly heterogeneous group of diseases that carry multiple somatic mutations and genomic structural changes. These findings have implications for therapeutic target identification and the design of future clinical trials for this aggressive group of breast cancer.

## 1. Introduction

Triple negative breast cancer (TNBC) is defined by the absence of estrogen receptor (ER), progesterone receptor (PR), and HER-2 Overexpression. It accounts for 15–20% of all breast cancer cases [1, 2], but occurs at a higher frequency in young premenopausal women with African Ancestry (AA) [3]. High body mass index (BMI) and high parity, instead of low parity in other types of breast cancer, have been associated with increased risk for TNBC [4–6]. TNBC is associated with an overall poor prognosis as exemplified by a higher rate of early recurrence and distant metastasis to brain and lungs compared to other breast cancer subtypes [7, 8]. The unfavorable clinical outcome is partly explained by its aggressive pathologic features including a higher histology grade and mitotic index [9]. 

Chemotherapy is the only systemic therapy currently available for TNBC and is curative in a subset of patients with chemotherapy-sensitive disease. A higher rate of pathologic complete response (pCR) to standard chemotherapy has been observed in patients with TNBC compared to ER+ disease. A pCR rate of 22% in TNBC versus 11% in ER+ disease was reported in a study of over 1 000 patients treated with neoadjuvant anthracycline and taxane-based chemotherapy regimens [10]. The excellent outcome associated with the pCR, however, is in contrast to the high risk of recurrence and cancer-related deaths in those with residue disease. Although alternative agents such as platinum compounds have demonstrated promising activity, up to 70–80% of patients have residual cancer following neoadjuvant cisplatin [11]. In the metastatic setting, TNBC is typically associated with an initially higher response rate, but in a shorter time to progression following treatment with existing chemotherapy agents, resulting a shorter overall survival compared to ER+ breast cancer in multiple studies [12]. The underlying molecular mechanism for this paradox is yet to be elucidated, although one could hypothesize that the inherent genomic instability of TNBC renders the possibility of a faster adaptation to the cytotoxic effect of chemotherapy.

The treatment options for chemotherapy-resistant TNBC are limited. The established targeted therapies, including endocrine treatment and HER2-targeted agents, are ineffective. Although several small molecule inhibitors and monoclonal antibodies against important cellular pathways have been tested in clinical trials, none has entered clinical practice due to limited efficacy. A better understanding of the underlying biology of TNBC is therefore needed to identify new therapeutic targets and to pinpoint which TNBC patients may benefit from them. Recent advances in microarray and DNA sequencing technologies have made it possible to analyze the tumor at the genomic level for therapeutic target discovery. These studies indicate that TNBC is a molecularly heterogeneous group of diseases with highly complex genomic aberrations. A further classification at the molecular level may be possible to facilitate drug development. In this paper, we will examine recent publications on the molecular basis of TNBC, with a particular focus on genomewide studies and their implication for future clinical trials.

## 2. Molecular Subclassification of TNBC Based on Gene Expression Profiling

In the seminal paper by Sørlie et al. breast cancer was subdivided into five intrinsic molecular subtypes, including luminal A, luminal B, HER-2 enriched, normallike, and basallike, based on hierarchical clustering analysis of approximately 500 genes (termed the intrinsic gene set because expression was not modulated by treatment) on a cDNA microarray study of 65 breast tumors obtained from 42 different individuals (Table 1) [8]. The term luminal A and luminal B subtypes was coined to reflect the presumed luminal epithelial cells origin of these cancers because of similarities in gene expression pattern and the expression of ER [13]. In contrast, HER-2-enriched subtype has high expression of HER-2 and genes that are close to HER-2 in the genome such as *GRB7*, but low expression of luminal and hormone receptor-related genes. Some of the clinical HER-2-positive cancers actually do not fall into HER-2-enriched subtype but belong to the luminal categories because of the coexpression of ER. These tumors are likely biologically different from those of the HER-2-enriched intrinsic subtype. Normal-like subtypes have, as their name implies, similar expression pattern to normal breast tissue. The significance of this subtype has yet to be determined, and some argue that it may represent a mere contamination of samples with normal breast tissue. The intrinsic subtypes carry prognostic significance with the basal-like subtype having the worse clinical outcome. The recent development of the 50-gene subtype predictor (PAM50), a RT-PCR assay that assigns intrinsic subtypes using RNA from formalin fixed and paraffin embedded tissue, has created a possible gold standard intrinsic subtype test for clinical application [14]. Although all intrinsic subtypes have been identified in TNBC, basal-like subtype is the most common, followed by the recently identified Claudin-low subtype [15].

### 2.1. Basal-Like Subtype

TNBC is most commonly associated with basal-like intrinsic subtype. Basal-like subtype is termed after the basal epithelial layer cells due to their similarities in gene expression pattern. Basal-like breast cancers typically express basal cytokeratins such as CK5/6, CK17 as well as cadherin, and epidermal growth factor receptor (EGFR) [16]. They are also frequently triple negative (negative for ER, PR, and HER2). In one study, about 70% of TNBC belonged to basal-like subtype, and 76% of basal-like cancers were found to be triple negative [1]. Many studies have used the two interchangeably, however, and it is important to note that although there is significant overlap, basal-like subtype does not encompass all of TNBC and may itself be another too broad of a classification. 

An association has been described between the basal-like subtype and *BRCA1*-gene-related breast cancers. The majority of *BRCA1*-related tumors are basallike by microarray analysis [12, 17], and sporadic basal-like breast cancers have been associated with “*BRCA*ness,” which is characterized by high tumor grade, lymphocytic infiltrate, pushing margins, ER and HER2 negativity, association with *TP53* mutations, c-myc amplification, and multiple chromosome abnormalities including X-chromosome isodisomy [18]. Although somatic mutations in *BRCA1/2* rarely occur in sporadic breast cancer [19–21], a rather high incidence, approaching 20%, of germ-line mutations in *BRCA1* or *2* has been reported in patients with TNBC [21]. In a study of 77 cases of sporadic TNBC from MD Anderson, *BRCA1 *mutation was identified in 12 (15.6%) (only one somatic) and *BRCA2 *mutation was identified in 3 (3.9%) [21]. More commonly, loss of *BRCA* expression due to gene silencing by promoter methylation has been shown in TNBCs [22]. It has been demonstrated that *BRCA1* normally suppresses the expression of basal-like-related genes, which could provide an explanation for “*BRCA*ness” of basal-like sporadic cancers [23].

The molecular similarity between basal-like sporadic breast cancers with *BRCA*-related cancers has raised the possibility and excitement that PARP inhibitors could be effective in this patient population. *BRCA1*/2 is important for homologous DNA repair. In the background of *BRCA* deficiency, DNA damage repair relies on alternative pathways such as base excision repair pathway provided by PARP, therefore inhibition of PARP would lead to accumulation of unrepaired DNA damage and cell death. This synthetic lethal approach has been shown to be effective in *BRCA*-related cancers in both preclinical and clinical settings [24–26]. The effectiveness of PARP inhibitors in TNBC, on the other hand, is not as clear. A recently published phase III trial of Iniparib in patients with TNBC did not yield positive results [27]. However Iniparib, which was originally thought to be a PARP inhibitor, turned out to have a more complicated mechanism of action unrelated to PARP so the results of this study may not be applicable to *bona fide* PARP inhibitors. The question also remains as to how to best identify patients who may benefit from these agents. In the absence of a robust biomarker predictor of treatment response, trials of PARP inhibitors in TNBC are being conducted in all comers rather than a defined molecular subtype.

### 2.2. Claudin-Low Subtype

Claudin-low is the latest subtype being identified by gene expression profiling studies [28]. It is characterized by the lack of expression of claudin proteins, which are important components of tight junctions that seal the potential space between adjacent epithelial cells, and epithelial cell adhesion molecules E-cadherin, EpCAM, and mucin-1 [15]. Claudin-low tumors are typically triple negative (61–71%), and conversely 25 to 39% of triple negative breast cancers are of the claudin-low subtype [15]. This subtype differs from the basal-like tumors, however, due to inconsistent expression of basal keratins and a significantly lower expression of proliferation genes [15]. They also have low expression of luminal markers, high expression of epithelial-to-mesenchymal transition (EMT) markers, and cancer stem-cell-like features. The expression of EMT markers is especially important in this group since it has been associated with resistance to therapy and higher metastatic potential [29, 30]. Claudin-low subtype accordingly was found to have a lower pathologic complete remission (pCR) rate with neoadjuvant chemotherapy than basal-like but higher than that of the luminal subtype putting their prognosis in between the two [15]. The identification of this subtype has provided further evidence of the broad underlying biology of TNBC and the need for a better understanding of the underlying biology of different subtypes of breast cancer and their therapeutic implications.

### 2.3. More Subtypes?

To specifically subclassify TNBC, Lehmann et al. analyzed the gene expression profile of 587 TNBC cases from 21 breast cancer databases and performed clustering analysis. Six subtypes were identified which may have therapeutic implications (Table 2) [31]. Two basal-like subtypes, BL1 and BL2, were the most prevalent and were so named because of their similarity to the previously described basal-like intrinsic subtype. These tumors have high expression of genes involved in cell cycle and cell division such as Aurora kinase and *MYC* and are highly proliferative as marked by high Ki-67 nuclear staining (BL1+BL2: 70% versus other subtypes: 42%). These results suggest that chemotherapies that target cell division and mitosis, such as taxanes, would be most applicable in this class. Indeed, BL1 and BL2 subtypes were associated with a significantly higher rate of pCR (63%; *P* = 0.042) with taxane-based therapies as compared to mesenchymal-like (31%) or luminal androgen receptor (14%) subtypes [32]. In addition, elevated expression of DNA damage response pathway genes such as *CHEK1* and *RAD51* were present in the BL1 subtype, and representative cell lines were found to be preferentially responsive to cisplatin which induces DNA damage [31]. 

A third subtype, immunomodulatory (IM), was found to be enriched in genes involved in immune processes. These include immune transduction pathways (NFKB, TNF, JAK), cytokine signaling such as IL-2 pathway, and antigen processing, among others. This subtype may represent medullary breast cancer, a subtype of TNBC that has a good prognosis, based on a similar expression profile reported in another study [33]. 

Mesenchymal (M) and mesenchymal stemlike (MSL) subtypes were characterized by expression of cell motility genes and proteins of the extracellular matrix. The MSL subtype displayed low expression of claudins 3, 4, and 7, consistent with the claudin-low subtype of breast cancer as previously discussed. MSL subtype also expressed genes involved in growth factor signaling such as EGFR and PDGFR pointing to possible therapeutic options in this subtype. Cell line models of M and MSL responded to inhibitors of PI3K/mTOR or Src.

The sixth subtype, luminal androgen receptor (LAR), was found to be enriched in genes involved in steroid synthesis and androgen metabolism. It has been reported previously that a proportion of TNBC may use or be dependent on the endocrine pathway despite being negative for ER and PR [34]. This was replicated in the study by Lehmann et al. in that a distinct subtype of TNBC, LAR subtype, was identified that has high expressions of hormonal related genes. Androgen receptor mRNA was expressed at an average of 9-fold higher level in this subtype than all the other subtypes [31]. Interestingly, LAR subtype belongs to either luminal A or luminal B intrinsic subtype despite being negative for ER expression. The finding of LAR subtype presents an exciting venue for endocrine treatment for at least a proportion of TNBC patients.

### 2.4. Subclassification of ER Breast Cancer Based on Kinase Gene Expression

In an attempt to identity kinase targets, Speers et al. investigated global kinase gene expression pattern and identified 52 kinases that are differentially expressed between ER-positive and ER-negative tumors [35]. The authors were able to further classify ER negative cancers into four types based on the expression of these kinases. One subtype was defined by the expression of cell cycle control kinases such as AK2, TTK, and CHK1. The second expressed kinases in the S6 pathway. Third subtype was defined by kinases involved in modulating the immune system such as LYN, IRAK1 and the fourth subtype defined by expression of MAPKs. Some of these tumors overexpressed HER-2 so this classification cannot be used specifically for TNBC, but these kinase-based subtypes may have therapeutic implications in targeting a particular pathway in TNBC.

## 3. Whole Genome Sequencing

The first comprehensive genomic analysis of a basal-like breast cancer was performed by using massively parallel sequencing technology and was published in 2010 [36]. The genome of the primary breast tumor obtained at initial diagnosis was compared with a brain metastasis developed at recurrence and a xenograft generated from the primary breast tumor in an immunodeficient mouse. Fifty novel somatic point mutations and small indels as well as 28 large deletions, 6 inversions, and 7 translocations were identified, including mutations in *TP53*, *JAK2*, and *MAP3K8,* among others. There was a wider range of mutation frequencies in the primary tumor compared to the brain metastasis and the xenograft, suggesting the existence of genetically heterogeneous tumor cell populations in the primary breast tumor that underwent clonal selection during the metastasis process and the generation of xenograft. Overall this basal-like breast cancer proved to possess an impressively complex genome. Compared to the genome of the two acute myeloid leukemia (AML) cases that were recently published, this basal-like cancer genome had 3- to 4-fold more single nucleotide variations (SNVs) [37, 38]. More genomic studies like this, however, are needed to create a genetic landscape of TNBC to guide therapeutics development. Importantly, as more genomic data is being generated, a significant challenge remains to differentiate “driver mutations” from “carrier mutations.” Individualized treatment would not be possible before we fully understand the biology of these genetic abnormalities.

## 4. Potential Therapeutic Targets for TNBC

### 4.1. SRC Inhibition

Src is a nonreceptor tyrosine kinase involved in cell adhesion and motility [39]. In preclinical studies, TNBC cell lines showed the highest sensitivity to dasatinib, a small molecule kinase inhibitor of Src, Abl, and KIT [31, 40]. Clinical studies have been disappointing however. A phase II trial of single-agent dasatinib in patients with advanced TNBC (CA180059) was reported in an abstract form. Dasatinib was found to have modest single-agent activity in these unselected TNBC patients with partial response in 5% and disease control rate in around 10% of 44 treated patients [41]. A smaller study using single-agent saracatinib, also a Src inhibitor, on nine ER/PR negative patients, failed to provide positive results [42]. A specific subtype of TNBC with Src dependence likely needs to be targeted to provide a benefit from this class of medications. Preclinical work indicates that the mesenchymal-like subtypes are more sensitive to Src inhibitors [31]. Mesenchymal-like subtypes are enriched in cell motility pathways, and Src is known to play an important role in cell migration likely explaining their sensitivity to this class of drugs.

### 4.2. PARP Inhibition

As mentioned earlier, PARP inhibitors have been an area of enthusiastic research in recent years for the treatment of TNBCs given their similarity to *BRCA*-related breast cancers. Promising results were found in a phase II trial of Iniparib in TNBC [38]. But as mentioned, the benefits were not confirmed in the subsequent phase III trial [27]. Given that the mechanism of action of Iniparib is now questioned and it is likely not a PARP inhibitor as originally thought, it is unclear what the implications of this study are for true PARP inhibitors. The PARP inhibitor Olaparib (AZD2281), which has been shown to be safe and effective in *BRCA*-related cancers, as well as other PARP inhibitors is currently being tested in clinical trials of TNBCs [26]. The results of these studies are eagerly awaited. Given the similarity of *BRCA*-related tumors and basal-like subtype, targeting this subtype of TNBC may provide the most benefit from these medications.

### 4.3. Androgen Receptor Inhibition

An interesting target that is currently under investigation is the androgen receptor (AR). As mentioned earlier, despite being negative for ER and PR, some TNBCs are positive for downstream targets of the endocrine pathway such as the androgen receptor. These cancers are likely concentrated in the LAR subtype of TNBC described by Lehmann et al.[31] and could be still dependent on an endocrine therapy responsive pathway. Although there have been no studies targeting this particular subtype with an AR inhibitor, a phase II trial (NCT00468715) is currently ongoing evaluating bicalutamide, a commonly used androgen receptor antagonist, in patients with ER/PR-negative breast cancer. If effective, this pathway has the potential to provide a nontoxic and targeted treatment strategy in this subtype of TNBC.

### 4.4. Targeting Epigenetics

There is evidence of gene silencing in patients with TNBC by methylation and/or histone acetylation [22]. In a recently published abstract on the first whole genome methylation analysis, TNBCs were indeed found to have a distinct methylation pattern from hormone receptor positive breast cancers [43]. Therefore it was hypothesized that epigenetic silencing may be involved in the lack of hormone receptors in TNBCs and demethylating or deacetylating agents could possibly reactivate genes involved in the endocrine pathway and subsequently restore sensitivity to endocrine therapy in these TNBCs. One study published in abstract form showed reexpression of ER and PR in TNBC after treatment with the combination of LBH589, a histone deacetylase inhibitor, and decitabine, a known hypomethylating agent [44]. There is another ongoing trial using single-agent decitabine in patients with TNBC followed by examination for ER expression and treatment with Tamoxifen (NCT01194908). It is not clear whether benefit from epigenetic manipulation would apply to all TNBCs or a specific subtype since gene methylation analysis was not tested in the published subtyping analysis.

### 4.5. EGFR Inhibition

Overexpression of EGFR is common in patients with TNBC and is seen in up to 60% of basal-like breast cancers [16]. It is associated with lower response to chemotherapy and poor overall survival [16]. As stated above, BL-2 and MSL subtypes of TNBC have been found to have higher expression of EGFR pathway genes. Trials have not targeted these specific subtypes with EGFR inhibition, and results have been disappointing in several published abstracts [45, 46]. In a study of 102 patients with TNBC, patients were randomized to weekly cetuximab plus or minus carboplatin at AUC of 2. Patients who received the combination therapy with carboplatin had a better response rate (18% versus 6%) and clinical benefit (PR or SD > 6 mo) of 27% versus 10% [45]. A second study tested carboplatin and irinotecan plus or minus cetuximab. Despite a higher response rate in the cetuximab-containing regimen (49% versus 30%), PFS was similar between the two groups (5.1 months versus 4.7 months) [46]. Activation of downstream EGFR targets, such as PI3K, may be responsible for limiting responses to EGFR directed therapy [47].

### 4.6. PI3K Pathway Inhibition

Phosphatidylinositol 3-kinase (PI3K), which is downstream of growth factor receptor signaling pathway, plays an important role in cell survival and proliferation and has been shown to be activated in a subset of TNBC, due to PTEN loss or less commonly *PIK3CA* mutation [48, 49]. Low PTEN expression was present in more than 60% of TNBC tumors in one study [48]. Since loss of PTEN is associated with increased activation of downstream Akt and predicts response to PI3K pathway inhibitors in preclinical models [50, 51], inhibitors of PI3K pathways could potentially have therapeutic efficacy in a subset of TNBC. For example, NVP-BEZ235, a PI3kinase inhibitor, has shown significant antitumor effect in the mesenchymal-like subtype of TNBCs, which are known to have higher expression of genes involved in EGFR pathway [31]. Clinical trials of PI3K pathway inhibitors are needed to confirm the preclinical findings.

## 5. Conclusions

Chemotherapy-resistant triple negative breast cancer remains a major cause of mortality and currently lacks any proven targeted therapy. The search for new therapeutic targets is complicated by the tremendous complexity of this disease, as demonstrated by the recent report of the first completed genome of a basal-like breast cancer. At the level of gene expression, the TNBC group also is actually comprised of distinct subtypes with very different biological signatures. All these subtypes would benefit from comprehensive analysis at the genomic, epigenomic, and proteomic levels and the results of the cancer genome atlas project are awaited with great interest.

There were more than 120 ongoing trials focusing on TNBC at the time of writing of this paper per clinicaltrials.gov. As stated above, potential targeted therapy can be applied to TNBC depending on the subtype (Table 3). However, most of the current trials are conducted in otherwise unselected patients and not directed by predictive biomarkers or mechanistic hypotheses. If this relatively large number of trials does not produce a breakthrough, we must rethink our investigational approach for this highly heterogeneous group of breast cancers. The development of “genome-first approaches” where patients are stratified upfront and prospectively placed into clinical trials designed to address the therapeutic hypotheses generated by analysis of individual tumor profiles is surely the most logical approach to consider.

## Figures and Tables

**Table 1:** Intrinsic subtypes of breast cancer.

Intrinsic subtypes of breast cancer	Characteristics
Luminal A	High level expression of ER and ER-associated genes, associated with a favorable clinical outcome.
Luminal B	Low level expression of ER and ER-associated genes, associated with a higher tumor cell proliferation rate and a worse clinical outcome compared to the luminal A subtype.
HER-2 Enriched	High level expression of HER2 and GRB7, associated with a poor outcome before the era of HER2-targeted agents.
Basal-like	Positive for the expression of basal cytokeratin but negative for the expression of luminal- and HER2-related genes, associated with a high tumor cell proliferation rate and a poor clinical outcome.
Normal-like	Similar expression compared to normal breast, suspicious for normal cell contamination.
Claudin-low	Lack the expression of claudin proteins that are implicated in cell-cell adhesion, but high expression of EMT and putative stem cell markers, associated with ER and HER2 negativity but low in basal cytokeratin expression.

**Table 2:** Six subtypes of triple negative breast cancer based on gene expression profiling.

Subtype	Gene expression profile
Basal-like 1 (BL-1)	High in the expression of genes involved in cell cycle progression, cell division, and DNA damage response pathways.
Basal-like 2 (BL-2)	High in the expression of genes involved in cell cycle progression, cell division, and growth factor signaling.
Immunomodulatory (IM)	High in the expression of genes involved in immune processes and cell signaling.
Mesenchymal (M)	High in the expression of genes involved in motility and extracellular matrix.
Mesenchymal stem-like (MSL)	High in the expression of genes involved in motility, extracellular matrix, and growth factor signaling; consistent with claudin-low Intrinsic subtype.
Luminal androgen receptor (LAR)	High in the expression of genes involved in hormonally regulated pathways.

**Table 3:** Potential Therapeutic Strategies and agent examples for TNBC.

Potential therapeutic strategies	Agent examples
Src inhibition	Dasatinib; Saracatanib
PARP inhibition	Olaparib; ABT-888
Androgen receptor inhibition	Bicalutamide
Targeting epigenetics	Decitabine LBH589
EGFR pathway inhibition	Cetuximab
PI3K pathway inhibition	NVP-BEZ235; Everolimus
